# Identification of Plant Growth Promoting Rhizobacteria That Improve the Performance of Greenhouse-Grown Petunias under Low Fertility Conditions

**DOI:** 10.3390/plants10071410

**Published:** 2021-07-09

**Authors:** Kaylee A. South, Nathan P. Nordstedt, Michelle L. Jones

**Affiliations:** Department of Horticulture and Crop Science, The Ohio State University, Wooster, OH 44691, USA; south.63@osu.edu (K.A.S.); nordstedt.1@osu.edu (N.P.N.)

**Keywords:** abiotic stress, biostimulants, *Caballeronia* *zhejiangensis*, floriculture, flower quality, greenhouse production, horticulture, nutrient stress, ornamentals, soilless media

## Abstract

The production of greenhouse ornamentals relies on high fertilizer inputs to meet scheduling deadlines and quality standards, but overfertilization has negative environmental impacts. The goals of this study were to identify plant-growth-promoting rhizobacteria (PGPR) that can improve greenhouse ornamental crop performance with reduced fertilizer inputs, and to identify the best measurements of plant performance for assessing the beneficial impact of PGPR on ornamentals. A high-throughput greenhouse trial was used to identify 14 PGPR isolates that improved the flower/bud number and shoot dry weight of *Petunia* × *hybrida* ‘Picobella Blue’ grown under low fertility conditions in peat-based media. These 14 PGPR were then applied to petunias grown under low fertility conditions (25 mg L^−1^ N). PGPR-treated plants were compared to negative (untreated at 25 mg L^−1^ N) and positive (untreated at 50, 75, 100, and 150 mg L^−1^ N) controls. Multiple parameters were measured in the categories of flowering, vegetative growth, and vegetative quality to determine the best measurements to assess improvements in ornamental plant performance. *Caballeronia zhejiangensis* C7B12-treated plants performed better in almost all parameters and were comparable to untreated plants fertilized with 50 mg L^−1^ N. Genomic analysis identified genes that were potentially involved in plant growth promotion. Our study identified potential PGPR that can be used as biostimulants to produce high-quality greenhouse ornamentals with lower fertilizer inputs.

## 1. Introduction

Greenhouse ornamental plants are typically grown in soilless, peat-based media with high fertilizer inputs to produce the highest quality crops in the least amount of time [1,2,3]. The marketability of ornamental plants is based on their visual attributes, which are important to both the grower and the consumer. Ornamental plant performance is based on growth, architecture, longevity, and quality, with the latter influenced by parameters such as flower and bud numbers, flower size and color, foliage color and shape, and the absence of pests and pathogens [4,5]. The production of high-quality ornamentals depends on environmental conditions and inputs that optimize plant nutrition and photosynthetic efficiency, while minimizing exposure to abiotic and biotic stresses.

Plants produced in containers of soilless media must acquire essential elements from fertilizer applications for proper crop growth and development [1,6,7]. Frequent fertilization is required due to the physical and chemical properties of soilless growing media that result in the reduced availability of applied nutrients [6,7,8,9]. Unlike soil-based production, plants grown in containers of soilless media have limited space for root expansion and limited volumes of growing media result in a lower buffering capacity and a limited nutrient supply [1,7]. In addition, applied nutrients may only be available to the plant for a short amount of time before they are leached out or become unavailable due to precipitation [7,9]. Depending on the growing media’s cation exchange capacity, the media may not be able to hold the nutrient ions for future use by the plant [9,10]. High fertilizer inputs are used to overcome these obstacles by promoting rapid vegetative growth and flower development during production; however, these plants are less tolerant of abiotic and biotic stresses, which can result in large crop losses during post-production shipping and retailing [3,4]. High fertilizer inputs are also a concern due to the cost and negative environmental impacts of fertilizer over-use [11,12,13].

The application of biostimulants can improve crop quality by promoting growth and improving stress tolerance, while minimizing chemical inputs. Microbial biostimulants containing mycorrhizal and non-mycorrhizal fungi, bacterial endosymbionts, and plant-growth-promoting rhizobacteria (PGPR) can increase nutrient use efficiency and improve nutrient acquisition when applied to plants during production [11,14,15,16]. This allows for the production of high-quality plants with reduced fertilizer inputs [17,18,19,20]. PGPR are beneficial bacteria that associate with the plant rhizosphere and improve plant performance through direct (e.g., increasing nutrient availability) or indirect (e.g., suppressing plant pathogens) mechanisms [21,22,23]. PGPR are an important tool for creating more sustainable agricultural systems [24]. For commercial containerized production systems that use soilless, peat-based media, many questions remain about how to efficiently utilize PGPR to improve plant performance [25].

PGPR can improve plant performance by increasing the availability, uptake, and use efficiency of essential nutrients [23,26]. Nitrogen-fixing bacteria are some of the most well-studied PGPR [23,27]. Many free-living bacterial species, including *Azospirillum* spp. and *Herbaspirillum* spp., which convert atmospheric nitrogen to ammonia, can increase nitrogen uptake by plants [23,28]. PGPR also include phosphate solubilizing bacteria that can facilitate the conversion of insoluble forms of phosphorus to soluble orthophosphates that are available for plant uptake [3,29,30]. Sulfur, like phosphorus, is predominantly inaccessible, and plants must rely on microbes to mobilize organically bound sulfur from sulfate-esters and sulfonates [31]. Many PGPR can also produce siderophores, which bind to ferric iron and increase iron availability to plants [27,32]. Plant-available forms of zinc can be limited, but PGPR can solubilize zinc and increase bioavailability [33,34,35,36].

Plant performance can also be improved by PGPR through the modulation of hormone levels [27,37]. The application of auxin-producing bacteria, such as *Bacillus licheniformis* and *Pseudomonas putida*, promotes fine root development, which increases nutrient uptake [38,39]. Many genera of PGPR including *Pseudomonas* and *Bacillus* produce the enzyme 1-aminocyclopropane-1-carboxylate (ACC) deaminase, which cleaves ACC, the direct precursor to ethylene. Reducing ethylene production by the plant also reduces the inhibitory effects of environmental stresses on root growth and development [21,40,41].

The application of beneficial bacteria can improve ornamental plant performance and nutrient use efficiency when applied to plants under low fertility conditions, but most of these reports are based on evaluations in soil [28,42,43,44]. Improvements in flowering parameters, such as flower diameter, are found in calla lilies (*Zantedeschia aethiopica*) grown in containers of sandy clay loam soil with 50% or 75% of the recommended phosphorus fertilizer rate and treated with a compost tea and a biofertilizer containing the PGPR *Enterobacter cloacae* [43]. Blanket flower (*Gaillardia pulchella*) seedlings treated with *Azospirillum* strains through a root dip and then grown in a raised nursery bed with 75% of the recommended fertilizer rate have shown higher flower yields and increased nitrogen uptake [28]. Improved flowering parameters and nitrogen uptake are also observed in petunia (*Petunia* × *hybrida*) grown with 50% of the recommended fertilizer rate and treated with *Azospirillum lipoferum* plus *Bacillus polymyxa* as a foliar spray at two growing stages, once 2 weeks after transplanting into a peat-mix media and again 3 weeks after repotting into soil-filled containers [44]. Ornamentals grown under greenhouse production conditions in soilless, peat-based media with frequent fertilization have different environmental and cultural conditions compared to plants that are grown under field conditions. These differences can impact the efficacy of PGPR application, and it is important to also evaluate PGPR under greenhouse production conditions [7].

The application of PGPR under typical greenhouse production conditions was explored by Nordstedt et al. [42], evaluating the application of individual PGPR to petunia, impatiens (*Impatiens walleriana*), and pansy (*Viola* × *wittrockiana*) plants grown in soilless media with a lower-than-recommended rate of water-soluble fertilizer at every irrigation (25 mg L^−1^ N). Nordstedt et al., [42] identified two *Pseudomonas* strains, originally isolated from a herbarium sample and Missouri soil, that increase shoot dry weight (DW) and the leaf nutrient content in petunia, impatiens, and pansy, as well as increasing the flower number in impatiens. PGPR that are isolated from the cropping system in which they are intended to be used may have an even greater effect on the promotion of plant growth than when the PGPR come from different cropping systems [28,45]. PGPR that are isolated from ornamentals produced in containers of peat-based media potentially have an even greater benefit on crop performance, and this led Nordstedt and colleagues [46] to create a collection of 1000+ bacteria isolated from the rhizosphere of commercial greenhouse-grown coleus (*Solenostemon scutellarioides*), petunia, geranium (*Pelargonium* × *hortorum*), vinca (*Catharanthus roseus*), and zinnia (*Zinnia elegans*).

A large portion of the screening for beneficial bacteria selection has been conducted in field crops, utilizing plant performance parameters that evaluate the qualities that are specific to these crops [47]. Emergence, yield, height, and shoot and root fresh weight (FW) and DW are common measurements used in evaluating field crop performance after PGPR application [45,48,49]. The plant performance parameters that are important for a high-quality ornamental crop are distinct from the parameters of field crops [4,5]. To select PGPR that are relevant to ornamental plant production, performance parameters that capture improvements in flowering, including open flowers and buds; vegetative size, which considers the canopy architecture; and vegetative quality, which considers the color of the leaves and stems, need to be evaluated.

There is a need to adopt new practices and technologies such as PGPR that have the potential to be used as biostimulants on greenhouse-grown ornamentals in soilless media to reduce fertilizer inputs and to give growers the tools needed to create a more sustainable production system [12,50]. In this study, 94 bacteria previously isolated from the rhizosphere of greenhouse ornamentals grown in soilless media [46] were evaluated for their ability to improve plant performance in petunias grown with reduced fertilizer inputs. The objectives of this study were (1) to select bacterial isolates from the greenhouse rhizosphere collection that increased the plant performance characteristics of petunias grown under low fertility conditions in soilless, peat-based growing media in a production greenhouse environment; (2) to identify the best plant performance characteristics that can be utilized in the selection of PGPR for ornamental greenhouse production; and (3) to use whole-genome sequencing to determine the taxonomic classification of the selected bacteria and gain insights into the putative mechanisms of plant growth promotion.

## 2. Results

### 2.1. High-Throughput Greenhouse Trials

**Figure 1:**
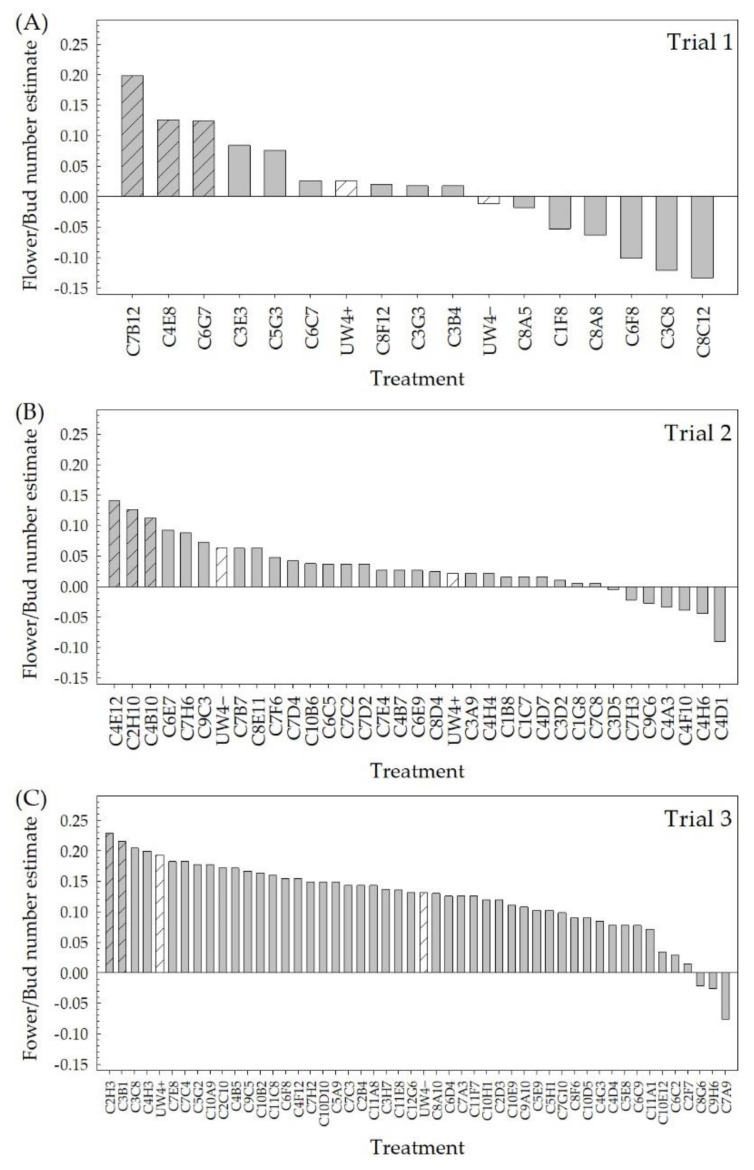
The number of flowers and buds (open flowers plus flower buds) of *Petunia* × *hybrida* ‘Picobella Blue’ treated with bacterial isolates from a greenhouse rhizosphere collection grown under low-fertility conditions (25 mg L^−1^ N) in the high-throughput greenhouse trials. Flower/bud number was counted 6 weeks after transplant. The 94 isolates were evaluated in three different groups, trial 1 (**A**), trial 2 (**B**), and trial 3 (**C**) (*n* = 12). Estimates of the treatment effect compared to the negative control (no bacterial treatment) from the Poisson or quasi-Poisson regression model were ranked. Positive values indicate strains that performed better than the negative control, whereas negative values indicate those that performed worse. *Pseudomonas putida* UW4+ and UW4− that were included as checks are represented by white bars with slanted lines. The top 10% of the total strains were then selected from this ranking (gray bars with slanted lines).

**Figure 2:**
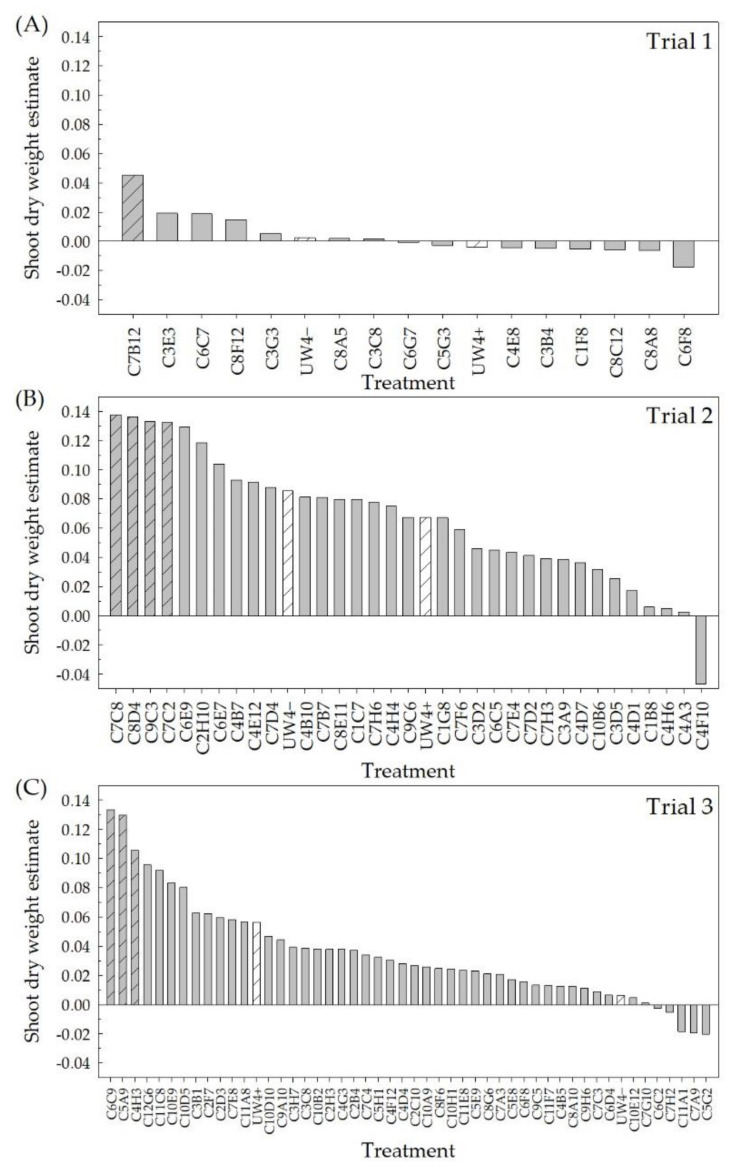
Shoot dry weight (DW) of *Petunia* × *hybrida* ‘Picobella Blue’ treated with bacterial isolates from a greenhouse rhizosphere collection grown under low-fertility conditions (25 mg L^−1^ N) in the high-throughput greenhouse trials. Shoots were harvested 6 weeks after transplant. The 94 isolates were evaluated in three different groups, trial 1 (**A**), trial 2 (**B**), and trial 3 (**C**) (*n* = 12). Estimates of the treatment effect compared to the negative control (no bacterial treatment) from the linear mixed-effects model were ranked. Positive values indicate strains that performed better than the negative control, whereas negative values indicate those that performed worse. *Pseudomonas putida* UW4+ and UW4−, which were included as checks, are represented by white bars with slanted lines. The top 10% of the total strains were then selected from this ranking (gray bars with slanted lines).

Ninety-four bacterial isolates previously selected from the greenhouse rhizosphere collection for the potential to alleviate water deficit stress in greenhouse ornamentals [46] were evaluated in high-throughput greenhouse trials for their ability to improve plant performance under low-fertility conditions. The estimates from the Poisson or quasi-Poisson regression of the flower/bud number and the fixed effect estimates of the treatment effects on the shoot DWs were used to select the top performing isolates from this group of 94 (Figure 1 and Figure 2). The positive values indicate isolates that had a higher flower/bud number or shoot DW compared to the negative control (no bacteria + 25 mg L^−1^ N fertilizer application), whereas the negative values indicate a lower flower/bud number or shoot DW compared to the negative control. The top 10% of the isolates in each of the three greenhouse trials for either flower/bud number or shoot DW were selected. From high-throughput greenhouse trial 1, C7B12 was selected for both increased flower/bud number (Figure 1A) and shoot DW (Figure 2A), whereas isolates C4E8 and C6G7 were selected based on increased flower/bud number (Figure 1A). Isolates C4E12, C2H10, and C4B10 were selected for increased flower/bud number from high-throughput greenhouse trial 2 (Figure 1B), and isolates C7C8, C8D4, C9C3, and C7C2 were selected for shoot DW (Figure 2B). In high-throughput greenhouse trial 3, C2H3 and C3B1 were selected for increased flower/bud number (Figure 1C), and isolates C6C9, C5A9, and C4H3 were selected for increased shoot DW (Figure 2C). Although check strains UW4+ and UW4− were not selected as top performing strains in either flower/bud number or shoot DW for any of the three trials, there were instances where these strains performed better than the negative control. When considering the flower/bud number, plants treated with UW4+ performed better than the negative control in all three trials and had increased shoot DW in trials 2 and 3. Plants treated with UW4− exhibited an increase in flower/bud number in trials 2 and 3 and an increase in shoot DW in all trials compared to the negative control.

The three high-throughput greenhouse trials identified 15 top bacterial isolates with the potential to promote growth and flowering. The sequence-based taxonomic classification of these top isolates identified strains from the following nine genera: *Caballeronia*, *Pseudarthrobacter*, *Raoultella*, *Pseudomonas*, *Curtobacterium*, *Herbaspirillum*, *Pantoea*, *Ochrobactrum*, and *Microbacterium* (Table 1). Sequencing identified that cultures of C3B1 contained more than one bacterial isolate, and it was therefore not included in the subsequent trials. Of the 14 strains selected for further evaluation, nine strains were isolated from various cultivars of coleus, two strains from zinnia, one strain from vinca, one strain from petunia, and one strain from geranium. These plants were collected from nine different greenhouse production facilities. Of the nine strains selected from coleus, three strains came from the same cultivar ‘Vino’ obtained from two different greenhouse production facilities (Table 1).

### 2.2. Greenhouse Validation Trial

**Figure 3:**
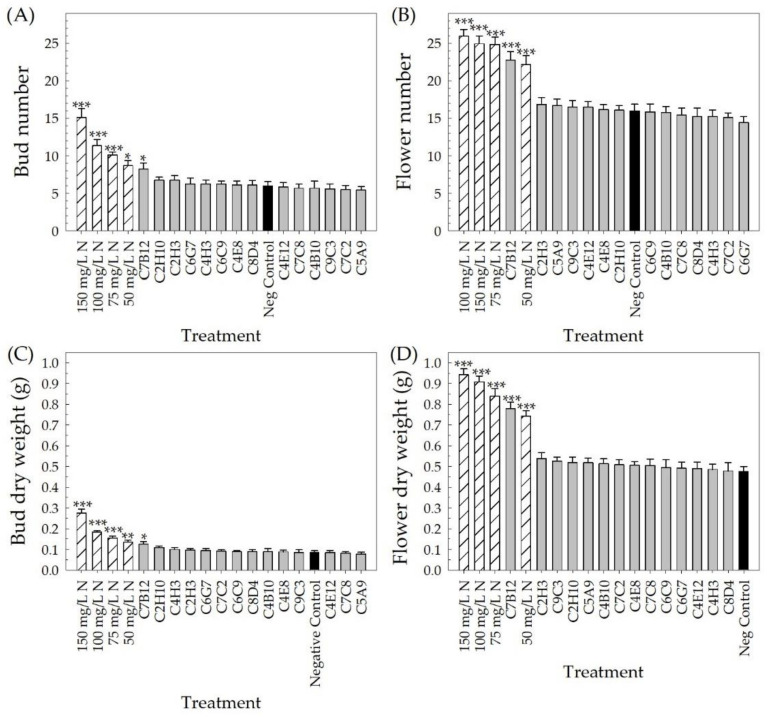
Flowering performance parameters measured in the greenhouse validation trial after application of the 14 bacterial strains selected from the high-throughput greenhouse trials to *Petunia* × *hybrida* ‘Picobella Blue’ (*n* = 12). Bud number (**A**) and flower number (**B**) were measured at harvest, 4 weeks after transplant, and bud dry weight (DW) (**C**) and flower DW (**D**) were measured after drying in a forced air oven (49 °C). All plants treated with bacteria were fertilized with 25 mg L^−1^ N (gray bars). The black bar represents the negative control (no bacterial treatment) fertilized with 25 mg L^−1^ N, and the white bars with slanted lines represent the four positive controls (no bacterial treatment) fertilized with either 50, 75, 100, or 150 mg L^−1^ N. The treatments were compared to the negative control using Dunnett’s comparison. Significance is represented by *, **, and *** at *p* ≤ 0.05, *p* ≤ 0.01, and *p* ≤ 0.001, respectively. Bars represent the mean, and error bars denote standard error.

**Flowering parameters**. The 14 strains selected from the high-throughput greenhouse trials were evaluated in a greenhouse validation trial under low-fertility conditions (25 mg L^−1^ N). Days to first flower, flower and bud number, and flower and bud DWs were used to evaluate flowering parameters. Neither the bacterial treatments (bacterial strain + 25 mg L^−1^ N) nor the positive fertilizer controls (no bacteria + 50, 75, 100, or 150 mg L^−1^ N) influenced the number of days to first flower (*p* ≥ 0.604) (data not shown). Plants treated with any one of the four positive fertilizer controls or C7B12 resulted in an increase in both bud and flower number compared to the negative control (*p* ≤ 0.040) (Figure 3A,B). The negative control plants had an average bud number of 6.0 ± 0.5, whereas the positive control plants had an average number of buds ranging between 15.1 ± 1.2 for 150 mg L^−1^ N control plants and 8.7 ± 0.7 for 50 mg L^−1^ N control plants. Similar to the 50 mg L^−1^ N control plants, treatment with C7B12 resulted in 8.3 ± 0.8 buds per plant (Figure 3A). Negative control plants had 16.0 ± 0.9 flowers per plant. The positive control plants had an average flower number ranging between 22.2 ± 1.2 for 50 mg L^−1^ N control plants and 25.9 ± 0.9 for 100 mg L^−1^ N control plants. Treatment with C7B12 resulted in 22.8 ± 1.1 flowers per plant (Figure 3B). Negative control plants had an average bud DW of 0.085 g ± 0.009 and flower DW of 0.477 g ± 0.023 per plant (Figure 3C,D). All positive fertilizer control plants and C7B12-treated plants had a higher bud DW (*p* ≤ 0.021) and flower DW (*p* < 0.001) compared to negative control plants. Plants receiving 150 mg L^−1^ N had the highest bud and flower DWs (0.275 g ± 0.020 and 0.942 g ± 0.029, respectively). Of the bacterial treatments, C7B12 resulted in the highest bud and flower DWs (0.125 g ± 0.013 and 0.779 g ± 0.030, respectively), which were comparable to the control plants that received 50 mg L^−1^ N (0.134 g ± 0.011 and 0.742 g ± 0.026, respectively) (Figure 3C,D).

**Figure 4:**
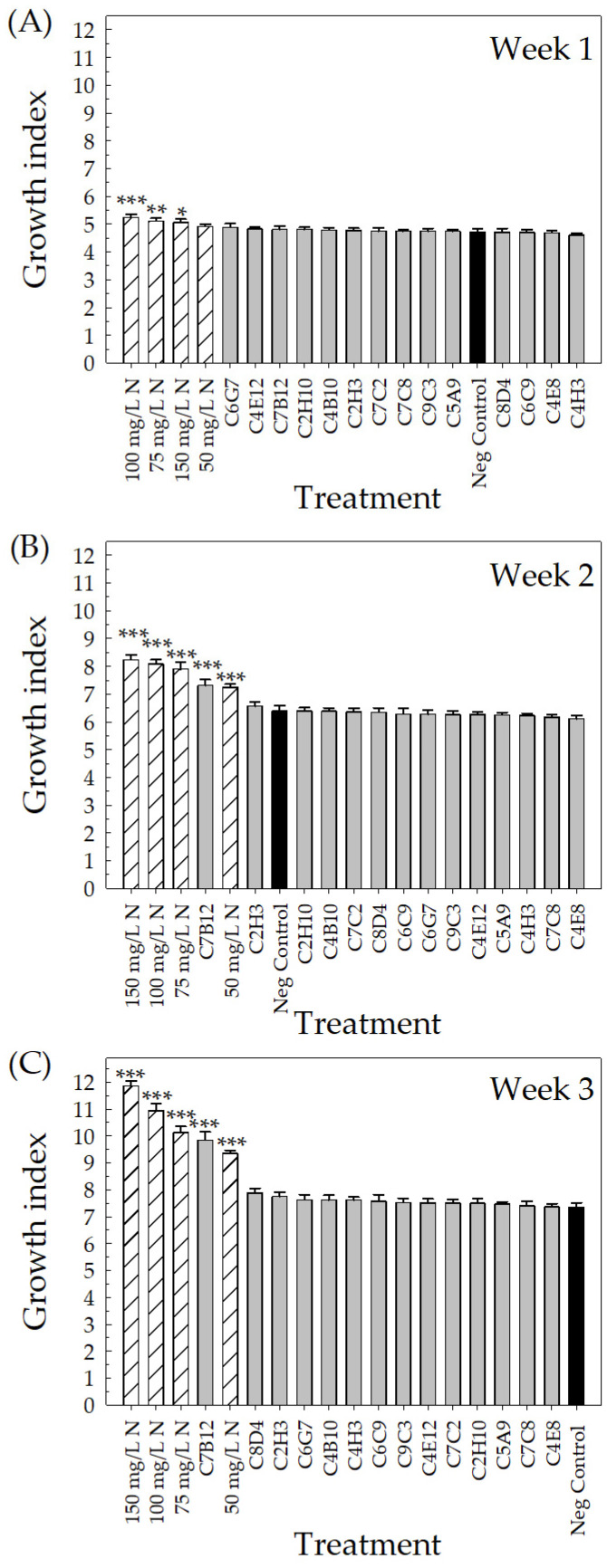
Growth index calculated from the plant height and two perpendicular widths of *Petunia* × *hybrida* ‘Picobella Blue’ in the greenhouse validation trial treated with 14 bacterial strains selected from the high-throughput greenhouse trials (*n* = 12). Growth index measurements are shown for one (**A**), two (**B**), and three (**C**) weeks after transplant. All plants treated with bacteria were fertilized with 25 mg L^−1^ N (gray bars). The black bar represents the negative control (no bacterial treatment) fertilized with 25 mg L^−1^ N, and the white bars with slanted lines represent the four positive controls (no bacterial treatment) fertilized with either 50, 75, 100, or 150 mg L^−1^ N. The treatments were compared to the negative control using Dunnett’s comparison. Significance is represented by *, **, and *** at *p* ≤ 0.05, *p* ≤ 0.01, and *p* ≤ 0.001, respectively. Bars represent the mean, and error bars denote standard error.

**Vegetative growth parameters.** Growth index, plant architecture rating, canopy cover, and shoot DW were used to evaluate the vegetative growth of the plants. In week 1, the growth index increased for control plants receiving fertilizer at 100 mg L^−1^ N (5.2 ± 0.1, *p* < 0.001), 75 mg L^−1^ N (5.1 ± 0.1, *p* = 0.002), and 150 mg L^−1^ N (5.1 ± 0.1, *p* = 0.013) compared to negative control plants receiving fertilizer at 25 mg L^−1^ N (4.7 ± 0.1) (Figure 4A). The four positive fertilizer controls and C7B12 (bacterial strain + 25 mg L^−1^ N)-treated plants had higher growth index values compared to the negative control plants (*p* < 0.001) in both week 2 and week 3. Control plants receiving 150 mg L^−1^ N had the highest growth index in week 2 (8.2 ± 0.2) and week 3 (11.9 ± 0.2) (Figure 4B,C). Negative control plants had a growth index of 6.4 ± 0.2 in week 2 and 7.4 ± 0.2 in week 3. Treatment with strain C7B12 resulted in a growth index of 7.3 ± 0.2 in week 2 and 9.9 ± 0.3 in week 3, which was similar to plants treated with 50 mg L^−1^ N in both week 2 (7.2 ± 0.1) and week 3 (9.4 ± 0.1) (Figure 4B,C).

**Figure 5:**
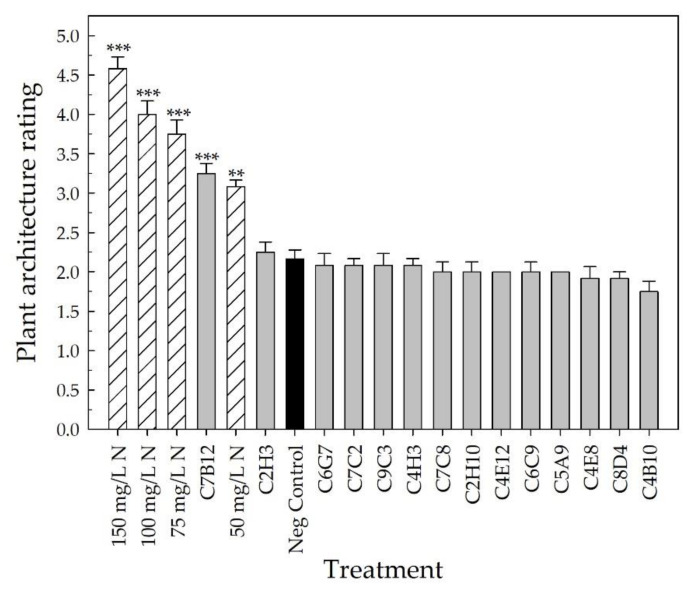
A plant architecture rating at harvest in the greenhouse validation trial was used to evaluate *Petunia* × *hybrida* ‘Picobella Blue’ treated with the 14 bacterial strains selected from the high-throughput greenhouse trials (*n* = 12). The rating scale ranged from 1 (vegetation did not cover the pot with ~40% of soilless growing media visible; plant appeared stunted) to 5 (vegetation covered pot fully; vegetation was not leggy). All plants treated with bacteria were fertilized with 25 mg L^−1^ N (gray bars). The black bar represents the negative control (no bacterial treatment) fertilized with 25 mg L^−1^ N, and the white bars with slanted lines represent the four positive controls (no bacterial treatment) fertilized with either 50, 75, 100, or 150 mg L^−1^ N. The plant architecture rating was analyzed using the Kruskal–Wallis rank sum test, followed by the Dunn’s test of multiple comparisons with FSA. Significance is represented by **, and *** at *p* ≤ 0.01, and *p* ≤ 0.001, respectively. Bars represent the mean, and error bars denote standard error.

**Figure 6:**
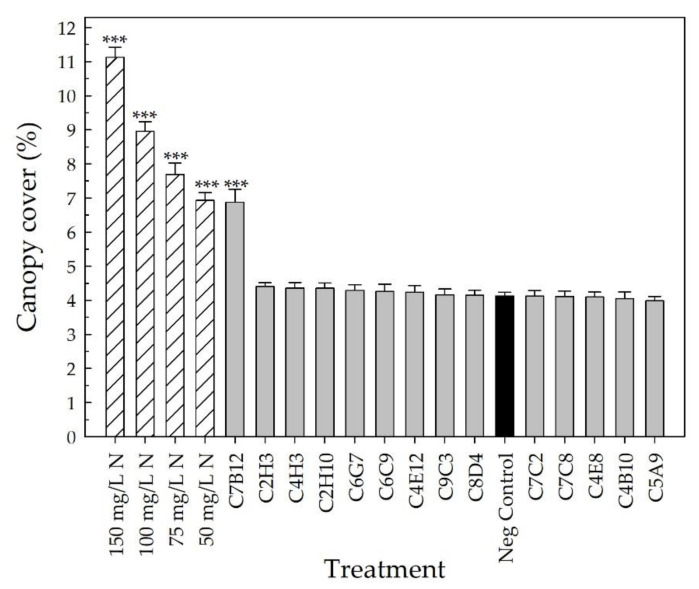
Percentage canopy cover of individual *Petunia* × *hybrida* ‘Picobella Blue’ in the greenhouse validation trial treated with the 14 bacterial strains selected from the high-throughput greenhouse trials (*n* = 12). Plants were measured at harvest after flowers and buds were removed. All plants treated with bacteria were fertilized with 25 mg L^−1^ N (gray bars). The black bar represents the negative control (no bacterial treatment) fertilized with 25 mg L^−1^ N, and the white bars with slanted lines represent the four positive controls (no bacterial treatment) fertilized with either 50, 75, 100, or 150 mg L^−1^ N. The treatments were compared to the negative control using Dunnett’s comparison. Significance is represented by *** at *p* ≤ 0.001. Bars represent the mean, and error bars denote standard error.

**Figure 7:**
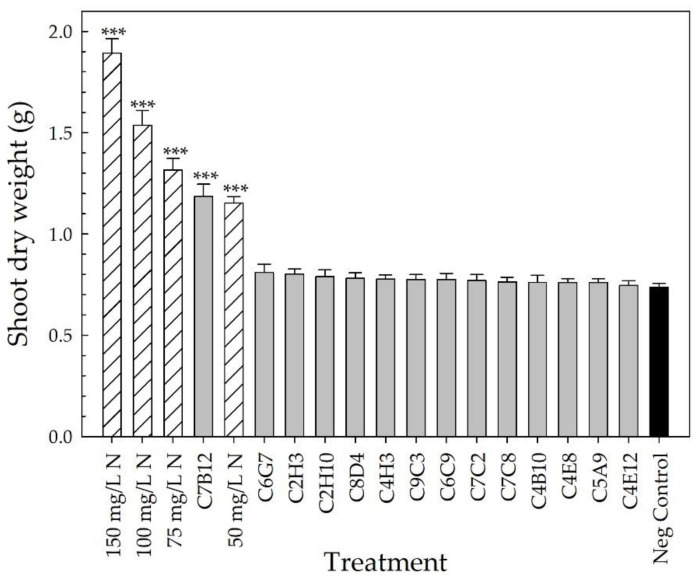
Shoot dry weight (DW) (g) was measured in the greenhouse validation trial of *Petunia* × *hybrida* ‘Picobella Blue’ treated with the 14 bacterial strains selected from the high-throughput greenhouse trials (*n* = 12). Shoots were harvested 4 weeks after transplant and dried in a forced air oven (49 °C). All plants treated with bacteria were fertilized with 25 mg L^−1^ N (gray bars). The black bar represents the negative control (no bacterial treatment) fertilized with 25 mg L^−1^ N, and the white bars with slanted lines represent the four positive controls (no bacterial treatment) fertilized with either 50, 75, 100, or 150 mg L^−1^ N. The treatments were compared to the negative control using Dunnett’s comparison. Significance is represented by *** at *p* ≤ 0.001. Bars represent the mean, and error bars denote standard error.

At harvest, the parameters of plant architecture rating, canopy cover, and shoot DW were used to evaluate vegetative growth. The plant architecture rating, which considered the fullness of the plant at harvest, showed that the four positive fertilizer controls resulted in an improvement in plant architecture compared to the negative control (*p* < 0.002) (Figure 5). Plants receiving 150 mg L^−1^ N had an average rating of 4.6 ± 0.1 (out of 5), whereas those receiving 50 mg L^−1^ N had an average of 3.1 ± 0.1. Strain C7B12 was the only bacterial treatment that resulted in a plant architecture rating that was better than the negative control (3.3 ± 0.1; *p* = 0.001). The average plant architecture rating of the C7B12-treated plants was similar to that of the 50 mg L^−1^ N control plants. The negative control plants had an average rating of 2.2 ± 0.1 (Figure 5). The four positive fertilizer controls had higher average canopy covers, with the range from 50 mg L^−1^ N plants averaging 6.9 ± 0.2% to 150 mg L^−1^ N plants averaging 11.1 ± 0.3% compared to the negative control plants (4.1 ± 0.1%, *p* < 0.001) (Figure 6). Strain C7B12 treated plants also had a higher canopy cover of 6.9 ± 0.4% (*p* < 0.001) compared to the negative control plants, which was again more similar to the 50 mg L^−1^ N control plants (Figure 6). The shoot DW was also used as a measure of the vegetative growth. The positive fertilizer controls continued to have higher averages compared to the negative control (*p* < 0.001), ranging from an average of 1.152 g ± 0.031 for 50 mg L^−1^ N plants to 1.893 g ± 0.072 for 150 mg L^−1^ N plants (Figure 7). The negative control plants had an average shoot DW of 0.737 g ± 0.019. As seen with the canopy cover, plants treated with C7B12 (1.185 g ± 0.061) had an increase in shoot DW (*p* < 0.001) that was more similar to 50 mg L^−1^ N treatment versus the negative control (Figure 7). As was seen with the flowering parameters, all other bacterial strains (except C7B12) showed a similar performance in all vegetative growth parameters to untreated controls that were similarly treated with 25 mg L^−1^ N.

**Figure 8:**
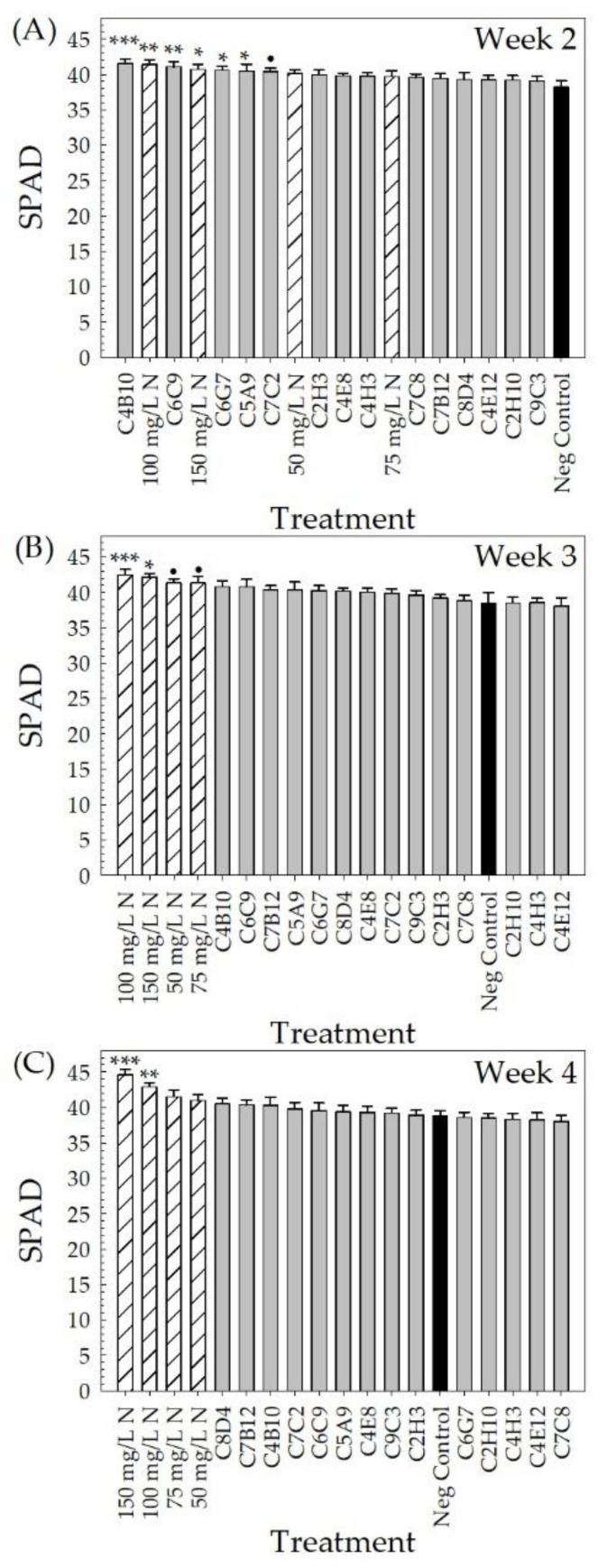
Relative chlorophyll concentration measured with the SPAD index in the greenhouse validation trial after application of the 14 bacterial strains selected from the high-throughput greenhouse trials to *Petunia* × *hybrida* ‘Picobella Blue’ (*n* = 12). SPAD index measurements are shown for two (**A**), three (**B**), and four (**C**) weeks after transplant. All plants treated with bacteria were fertilized with 25 mg L^−1^ N (gray bars). The black bar represents the negative control (no bacterial treatment) fertilized with 25 mg L^−1^ N, and the white bars with slanted lines represent the four positive controls (no bacterial treatment) fertilized with either 50, 75, 100, or 150 mg L^−1^ N. The treatments were compared to the negative control using Dunnett’s comparison. Significance is represented by **•**, *, **, and *** at *p* ≤ 0.1, *p* ≤ 0.05, *p* ≤ 0.01, and *p* ≤ 0.001, respectively. Bars represent the mean, and error bars denote standard error.

**Figure 9:**
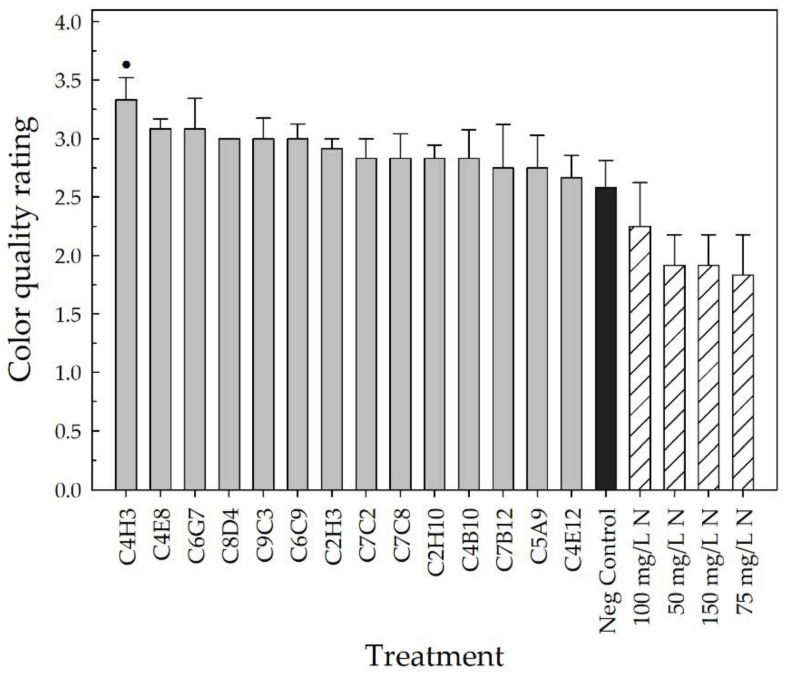
The color quality rating, which measured the visual color quality of the vegetation in the greenhouse validation trial, was evaluated at harvest of *Petunia* × *hybrida* ‘Picobella Blue’ treated with the 14 bacterial strains selected from the high-throughput greenhouse trials (*n* = 12). The rating scale ranged from 1 (interveinal chlorosis on five or more leaves) to 4 (good green vegetation with no discoloration). All plants receiving bacterial treatments were fertilized with 25 mg L^−1^ N (gray bars). The black bar represents the negative control (no bacterial treatment) fertilized with 25 mg L^−1^ N, and the white bars with slanted lines represent the four positive controls (no bacterial treatment) fertilized with either 50, 75, 100, or 150 mg L^−1^ N. The color quality rating was analyzed using the Kruskal–Wallis rank sum test, followed by Dunn’s test of multiple comparisons with FSA. (**•**) shows differences in color quality rating compared to the negative control at *p* ≤ 0.1. Bars represent the mean, and error bars denote standard error.

**Vegetative quality parameters.** The vegetative quality of the plants was monitored by measuring the relative chlorophyll concentration (SPAD index) of the leaves using a soil-plant analysis development (SPAD) meter (SPAD-502, Konica Minolta Sensing, Inc., Osaka, Japan). Neither the positive fertilizer controls (no bacteria + 50, 75, 100, or 150 mg L^−1^ N) nor the bacterial treatments (bacterial strain + 25 mg L^−1^ N) were different from the negative controls (no bacteria + 25 mg L^−1^ N) in week 1 (*p* > 0.164) (data not shown). In week 2, the negative control plants had the lowest SPAD index of 38.3 ± 0.8. C4B10-treated plants had the highest SPAD index of 41.5 ± 0.7, which was different from that of the negative control (*p* < 0.001) (Figure 8A). Plants treated with strains C6C9, C6G7, C5A9, or C7C2 (SPAD of 41.1 ± 0.7, 40.6 ± 0.5, 40.5 ± 1.0, and 40.4 ± 0.4, respectively) also had SPAD indices higher than those of the negative control plants (*p* ≤ 0.054). Similarly, positive control plants fertilized with 100 mg L^−1^ N had a SPAD index of 41.4 ± 0.7, and 150 mg L^−1^ N fertilized plants had an average of 40.7 ± 0.7 (*p* ≤ 0.020) (Figure 8A). In week 3, plants receiving 100, 150, 50, and 75 mg L^−1^ N had an increase in the SPAD index (42.5 ± 0.8, 42.2 ± 0.5, 41.4 ± 0.6, and 41.4 ± 0.9, respectively) compared to the negative control (38.6 ± 1.4, *p* ≤ 0.090) (Figure 8B). In week 4, plants receiving 150 mg L^−1^ N had the highest SPAD index (44.6 ± 0.8), followed by plants receiving 100 mg L^−1^ N (42.9 ± 0.6), both with increases over the negative control plants (38.8 ± 0.7, *p* ≤ 0.007) (Figure 8C). Vegetative quality was evaluated at harvest using a color quality rating. Although all bacterial treatments resulted in a higher color quality rating than the negative and positive control plants, only C4H3-treated plants were significantly higher than the negative control. Strain C4H3 had an average rating of 3.3 ± 0.2 (out of four), compared to the negative control plants with an average rating of only 2.6 ± 0.2 (*p* = 0.076) (Figure 9).

For each parameter, the treatments were ranked based on estimates or *z*-scores (for rating data) of the treatment effect to identify bacterial strains (bacterial strain + 25 mg L^−1^ N) that resulted in small improvements in plant performance compared to the negative control (no bacteria + 25 mg L^−1^ N). These rankings from each parameter were used to select the overall top-performing strains and to determine which strains resulted in improved plant performance in each performance category (i.e., flowering, vegetative growth, and vegetative quality) (Table 2). The estimates of treatment effects from the Poisson regression were ranked compared to the negative control for bud and flower number. In addition to C7B12, strains C2H10, C2H3, C6G7, C4H3, and C6C9 were also selected for enhanced flowering, with an average number of buds per plant of 6.8 ± 0.4, 6.8 ± 0.6, 6.3 ± 0.8, 6.3 ± 0.5, and 6.3 ± 0.4, respectively. Strains C2H3, C5A9, C9C3, and C4E12 were selected based on increased average flower numbers of 16.8 ± 0.9, 16.7 ± 0.9, 16.5 ± 0.9, and 16.5 ± 0.7, respectively. Fixed-effect estimates of treatment effects were ranked from the highest to lowest for bud and flower DWs. Strains C2H10, C4H3, and C2H3 were also selected for increasing bud DW (0.110 g ± 0.006, 0.100 g ± 0.010, and 0.096 g ± 0.009, respectively). For flower DW, C2H3, C9C3, and C2H10 were selected, with weights of 0.539 g ± 0.029, 0.525 g ± 0.020, and 0.519 g ± 0.025, respectively.

The bacterial treatments were ranked from highest to lowest for growth index, canopy cover, plant architecture rating, and shoot DW in the vegetative growth performance category and for SPAD and color quality rating in the vegetative quality performance category to select the top-performing strains. Due to the very small growth index differences between the negative and positive controls, week 1 was not considered in the identification of the overall top-performing bacterial strains. As stated above, C7B12 was a top performer in all vegetative growth parameters. Additional strains were identified in the vegetative growth performance category based on the rankings. In week 2, C2H3 (6.6 ± 0.1) was selected, and in week 3, C8D4 (7.9 ± 0.2), C2H3 (7.8 ± 0.2), and C4H3 (7.6 ± 0.1) were selected based on improvements in plant growth index. Strains C2H3 (4.4 ± 0.1%), C4H3 (4.4 ± 0.2%), and C2H10 (4.4 ± 0.2%) were selected as the top four bacterial treatments based on canopy cover rankings. In the case of the plant architecture rating, C3H3 (2.3 ± 0.1) was selected in addition to C7B12. Strains C6G7, C2H3, and C2H10 were selected based on the shoot DW rankings (0.809 g ± 0.041, 0.802 g ± 0.025, and 0.789 g ± 0.032, respectively). SPAD indices taken 2 weeks after transplant (week 2) through 4 weeks after transplant (week 4) were used to select bacterial strains that improved plant performance. As pointed out previously, C4B10, C6C9, C6G7, C5A9, and C7C2 were selected from week 2 as top performers, and C4B10 continued to be ranked among the top strains for week 3 and week 4 (40.9 ± 0.8 and 40.3 ± 1.1, respectively). Strains C6C9 (40.8 ± 1.1), C7B12 (40.4 ± 0.5), C5A9 (40.4 ± 1.1), and C6G7 (40.3 ± 0.7) were all selected as top performers based on their ranking of the fixed effect estimates in week 3, and strains C8D4 (40.5 ± 0.8), C7B12 (40.3 ± 0.7), and C7C2 (39.7 ± 1.0) in week 4. For the color quality rating, in addition to C4H3 as described previously, C4E8 (3.1 ± 0.1), C6G7 (3.1 ± 0.3), C8D4 (3.0 ± 0.0), C9C3 (3.0 ± 0.2), and C6C9 (3.0 ± 0.1) were selected (Table 2).

**Figure 10:**
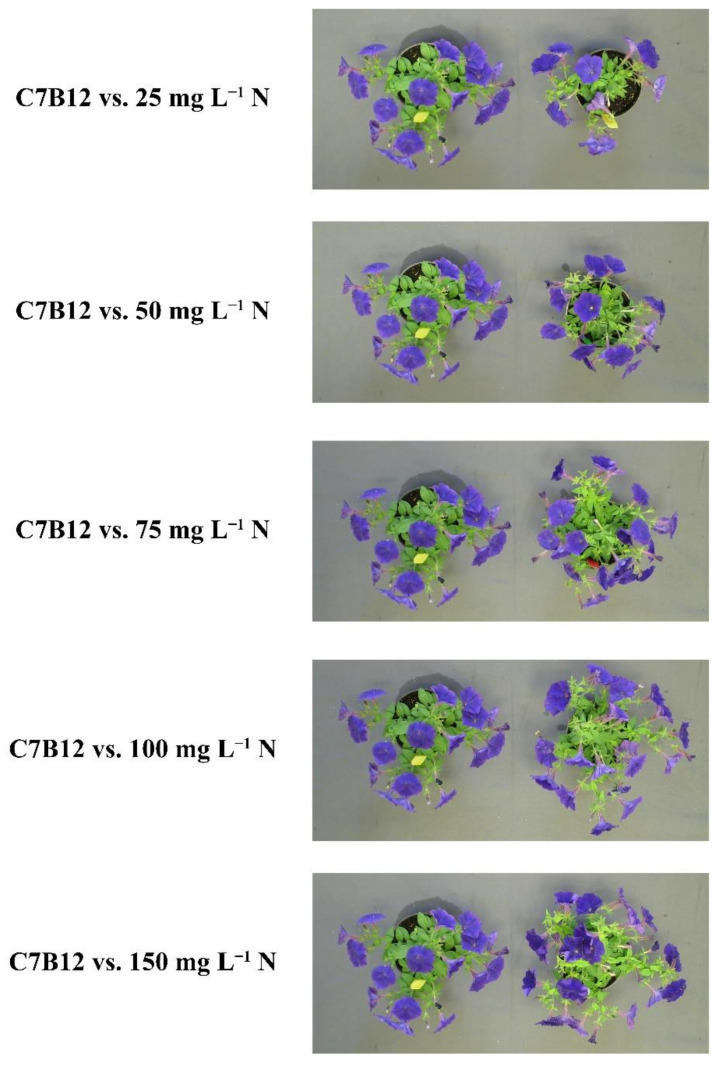
Visual quality of *Petunia* × *hybrida* ‘Picobella Blue’ treated with bacterial strain C7B12 and the fertilizer controls (no bacterial treatment). Plants treated with C7B12 were fertilized with 25 mg L^−1^ N at each irrigation. The negative control plants were fertilized with 25 mg L^−1^ N, and the four positive fertilizer controls were fertilized with 50, 75, 100, or 150 mg L^−1^ N (no bacterial treatment).

Strain C7B12 was the top performing strain in all performance categories. It was identified as one of the highest-ranked strains in all parameters except for SPAD index in week 2 and color quality rating. Plants treated with bacterial strain C7B12 were visually larger than the negative control (no bacteria + 25 mg L^−1^ N) plants, and they were comparable in size and quality to positive control plants receiving higher fertilizer rates (Figure 10). C7B12-treated plants also had less leaf yellowing compared to plants fertilized at higher rates. For these reasons, C7B12 was considered the top-tier strain. Strain C2H3, identified as a second-tier strain, was a top performer in most parameters except SPAD index and color quality rating. Four strains (C4H3, C2H10, C6G7, and C6C9) were identified within a third tier of strains based on their overall performance score. Strains C4H3 and C6G7 were selected for at least one parameter in all performance categories. Strain C2H10 was selected for several parameters in the flowering and vegetative growth performance categories, and C6C9 was selected for at least one parameter in the flowering and vegetative quality performance categories. Some strains performed well within specific performance categories. For instance, C9C3 was selected for both flower number and flower DW (i.e., flowering), and C4B10 was selected as a top performer in all three weekly SPAD index measurements (i.e., vegetative quality), although it was not selected for the color quality rating. Strain C2H10 was selected as a top performer for bud number and bud DW. Within the vegetative growth category, some strains were selected as top performers in all parameters, such as C7B12 and C2H3, but others performed well in only one or two of the parameters. This is true for C4H3, which was selected as a top performer in week 3 growth index and canopy cover, but not in the other vegetative growth parameters (Table 2).

### 2.3. Putative Plant-Growth-Promoting Mechanisms

The genomic sequences for the overall top-performing strains, C7B12, C2H3, C4H3, C2H10, C6G7, and C6C9, were used to identify genes potentially involved in plant growth promotion. These included genes involved in mineral nutrient transport, nitrogen and sulfur metabolism, phosphate and zinc solubilization, siderophore production, indole-3-acetic acid (IAA) synthesis, and ACC degradation (Table 3). Notably, none of the top strains contained *nif* genes, which are responsible for nitrogenase synthesis or nitric oxide reductase; however, all the top strains except C2H10 and C6C9 contained the *amtB*-*glnK* complex involved in ammonium transport. Genomes for strains C7B12, C2H3, C4H3, and C6C9 contained both *pqq* and *gdh* genes, which are involved in gluconic acid production, an indicator of phosphate solubilization capabilities. In addition, C7B12, C2H3, and C4H3 contained the complete gene cluster encoding the high affinity phosphate transport system (*pstABCS*). Strains C2H3 and C6G7 contained the sulfate ABC transporter complex (*cysPWAT-sbp*), as well as *cysND*, *cysC*, and *cysHIJ*, involved in sulfur metabolism. AntiSMASH analysis determined that all strains except C6C9 contained biosynthetic gene clusters (BGCs) for various siderophores. This included the siderophore BGCs for the synthesis of ornibactin (C7B12), turnerbactin (C2H3, C4H3), pyoverdin (C2H3), desferrioxamine (C2H10), and enterobactin (C6G7). All six strains except for C6C9 encoded for *ghrB,* which indicates that they can convert gluconic acid to 5-ketogluconic acid, an organic acid potentially involved in zinc solubilization [51,52]. Strains C2H3 and C4H3 contained genes encoding for a high affinity ABC transporter system (*znuABC*), and all strains except C4H3 contained genes that encode for zinc export (*zitB* or *zntABR*). The genome of C2H3 contained genes for the tryptophan (Trp) biosynthesis operon (*trpABCDE*), genes for the Trp-specific importer (*mtr*), and genes for one IAA synthesis pathway (*ipdC*), suggesting that it is capable of synthesizing IAA and its precursor Trp. Strains C7B12 and C4H3 both contained the ACC deaminase gene (*acdS*) (Table 3).

## 3. Discussion

Microbial biostimulants provide the greenhouse industry with more sustainable alternatives to chemical fertilizers, and their adoption by greenhouse growers can decrease the costs and environmental impacts associated with the excessive application of fertilizer nutrients to soilless growing media. Plant-growth-promoting rhizobacteria (PGPR) can improve plant performance under lower fertility conditions, allowing for the production of high-quality crops with reduced fertilizer inputs [17,23]. In this study, we selected bacteria isolated from the rhizosphere of greenhouse-grown ornamentals that could improve plant performance under low-fertility conditions, determined their taxonomic classification, and identified genes that could potentially be involved in promoting plant growth. We also described parameters in the categories of flowering, vegetative growth, and vegetative quality that are relevant to improved plant performance in ornamental plants that allowed for the selection of PGPR. The overall top-performing strains identified from these experiments were *Caballeronia zhejiangensis* C7B12, *Pantoea dispersa* C2H3, *Pseudomonas oryzihabitans* C4H3, *Curtobacterium* sp. C2H10, *Raoultella terrigena* C6G7, and *Ochrobactrum* sp. C6C9.

Of the 14 strains selected from the high-throughput greenhouse trials, *C. zhejiangensis* C7B12, isolated from coleus, was the only strain selected for both increased flower/bud number and shoot DW in the initial greenhouse trials (Table 1). In the greenhouse validation trial, plants treated with *C. zhejiangensis* C7B12 performed well in all performance categories, were statistically different from the negative control (no bacteria + 25 mg L^−1^ N) plants in the majority of parameters, and in many cases, performed more like plants grown with higher levels of fertilizer. In fact, the application of *C. zhejiangensis* C7B12 to plants fertilized with only 25 mg L^−1^ N resulted in improved or similar performance in each category to that of plants fertilized with twice as much fertilizer (50 mg L^−1^ N). The application of *C. zhejiangensis* C7B12 could allow for reduced fertilizer rates, while maintaining the desired quality of the plant. The genus, *Caballeronia*, came out of a regrouping of the *Burkholderia* genus that contains known PGPR, and *Caballeronia* now contains plant-associated, non-pathogenic strains [53,54,55]. One species, *Caballeronia sordidicola*, isolated from spruce trees (*Picea glauca ×* *engelmannii*) in low-fertility soils, shows potential plant-growth-promoting mechanisms in vitro such as IAA production and phosphate solubilization, and its application improved the plant growth parameters of spruce and pine (*Pinus contorta*) tree seedlings [56]. *Caballeronia zhejiangensis* is a species that has not been well studied. It was reported previously to be isolated from a waste-water treatment system [57] and agricultural soil [58,59]. In both instances *C. zhejiangensis* was found to degrade the pesticide methyl parathion and its main product after hydrolysis (p-nitrophenol) when grown in methyl parathion-amended culture medium [57,58], and Popoca-Ursino et al., [58] confirmed the presence of the genes involved in the degradation. There are no reports of this bacterial species being applied to plants for the purpose of plant growth promotion, nor on the potential mechanisms by which this plant growth promotion might occur.

Hoda and Mona [44] also found that petunias grown with 50% of the recommended fertilizer rate treated with beneficial bacteria (*Azospirillum lipoferum*, *Bacillus polymyxa*, or their combination) showed improved or similar plant heights, flower opening dates, numbers of flowers per branch, flowering periods, and leaf nitrogen contents compared to control plants receiving the full fertilizer rate; however, the bacteria were applied through spray application at two time points, the plants were grown partly in soil, and there was a one-time fertilizer application [44]. In our study, a weekly media drench was used as the PGPR application method since the bacteria of interest were isolated from the rhizosphere, [46] and this is a common application method for microbial biostimulants [15]. It is typical in greenhouse ornamental production to have a liquid fertilizer application at each irrigation [7] and to grow plants in peat-based soilless growing media, [6] both of which have an impact on the microbial community in the rhizosphere [8]. To select the best PGPR for use in the production of ornamentals, we simulated environmental and cultural practices typically found in this greenhouse production system.

*Pantoea dispersa* C2H3, isolated from zinnia, had the second highest overall score in the greenhouse validation trial and was considered a second-tier overall performing strain based on its high rankings in all flowering and vegetative growth parameters (Table 2). Vegetative growth promotion by *P. dispersa* has previously been reported in other plant species. A *P. dispersa* strain isolated from wheat increased shoot DW and zinc uptake in 3-month-old wheat plants [60]. Furthermore, a strain from the rhizospheric soil of mung beans identified as *P. dispersa* increased the shoot and root FW and DW when applied by means of seed inoculation and later through foliar spray application at flowering to mung beans grown in sterile soil [61]. In our study, we found that media drench applications of *P. dispersa* C2H3 similarly improved vegetative growth characteristics such as shoot DW. Our results also provide new insights into the growth promotion effects of *P. dispersa* on flower production, increasing both total flower number and DW.

*Pseudomonas oryzihabitans* C4H3, *Curtobacterium* sp. C2H10, *Raoultella terrigena* C6G7, and *Ochrobactrum* sp. C6C9 all had similar overall scores in the greenhouse validation trial, identifying a third tier of overall top-performing strains with more limited effects on plant responses in only some of the categories of vegetative and flowering performance. Interestingly, all four of these strains were initially isolated from coleus. Although there are limited reports of growth-promoting bacteria from the genera *Curtobacterium, Raoultella,* and *Ochrobactrum,* the pseudomonads are some of the most commonly identified and characterized PGPR [23].

All four strains resulted in some vegetative growth improvements, and all four strains except *Curtobacterium* sp. C2H10 resulted in vegetative quality improvements. Improvements in root and shoot length were previously observed in pearl millet (*Pennisetum glaucum*) grown in sterile soil treated with *P. oryzihabitans* [48], and wheat and barley (*Hordeum vulgare*) treated with *R. terrigena* in combination with boron showed improvements in root DW, shoot DW, and leaf total chlorophyll content [62]. Improvements have also been found with the application of some of these PGPR under abiotic stress. A legume, *Sulla carnosa*, under salt stress treated with *Curtobacterium* sp. showed an increased total dry biomass and total chlorophyll content [63], and corn plants under water stress treated with *Ochrobactrum* sp. showed improvements in many vegetative growth parameters, including shoot and root DW, in addition to improvements in total chlorophyll content [64]. Although improvements in vegetative growth and quality have been previously found with each of these strains, there are limited reports of plant performance improvements when grown under low nutrient stress or greenhouse production conditions.

All four of the third-tier strains were also selected for at least one flowering parameter. *Curtobacterium herbarum* applied to saffron crocus (*Crocus sativus*) did not increase flower number compared to the control but did increase saffron thread length (obtained from the flower) and saffron yield [65]. In our study, petunias treated with *Curtobacterium* sp. C2H10 showed a slight increase in flower and bud DW and bud number, but not in flower number. There are no reports of flowering parameter improvements in greenhouse-grown ornamental plants for *P. oryzihabitans*, *Curtobacterium* sp., *R. terrigena*, or *Ochrobactrum* sp.

The whole-genome sequences of the top six strains were used to identify the putative mechanisms responsible for the plant growth promotion that was observed in this study (Table 3). Beneficial bacteria can promote plant growth by facilitating increased nutrient uptake and by modulating phytohormone levels. PGPR can improve nutrient bioavailability and plant uptake via nitrogen fixation, nutrient solubilization, nutrient oxidation, and metal chelation [23,66].

Nitrogen is an essential plant macronutrient, which is made bioavailable in the form of nitrate and ammonium via both symbiotic and non-symbiotic bacterial nitrogen fixation [67]. None of the top strains identified in this study contained the genes encoding for the nitrogenase enzyme needed for nitrogen fixation, nitrate/nitrite ABC transporters, or nitrite reduction, but genes that are involved in nitrogen metabolism and ammonium, nitrate, and nitrite transport were found. Previously, some of these bacterial species have been shown to improve nitrogen content in plants. The application of a strain of *P. dispersa* to wheat seedlings grown at sub-optimal temperatures increases the nitrogen content of the seedlings [68], and a strain of soil-borne *Ochrobactrum* sp. increases the nitrogen content in common bean (*Phaseolus vulgaris*) [69].

The bioavailability of some essential nutrients, including phosphorus and zinc, depends on their solubility [29,33,35]. *Caballeronia zhejiangensis* C7B12, *P. dispersa* C2H3, *P. oryzihabitans* C4H3, and *Ochrobactrum* sp. C6C9 all contained the genes *pqq* (pyrroloquinoline quinone synthase) and *gdh* (glucose dehydrogenase), which are involved in the production of gluconic acid, a common mechanism for phosphate solubilization [70,71,72]. Of those strains, *Caballeronia zhejiangensis* C7B12, *P. dispersa* C2H3, and *P. oryzihabitans* C4H3 also contained genes encoding the high affinity phosphate transporter system (*pstABCS*), which is involved in the uptake of phosphorus [73,74] (Table 3). There are reports of bacteria within these genera that can increase the bioavailability of phosphorus. A *P*. *dispersa* strain isolated from cassava (*Manihot esculenta*) can solubilize phosphorus in vitro and in soil [75]. In vitro phosphate solubilization was also reported for a *P. oryzihabitans* strain isolated from maize [76] and *Ochrobactrum* sp. strains isolated from soil [69,77]. All the top six bacterial strains had some genes involved in zinc solubilization and/or transport. Notably, *P. dispersa* C2H3 was the only strain that encoded the high affinity ABC zinc transporter system and the other three genes identified as being important for bacterial solubilization and the transport of zinc. *Pantoea dispersa* has been reported to solubilize zinc in vitro and increase shoot zinc content when applied to wheat [60].

Organic forms of sulfur are converted to plant bioavailable forms such as sulfate through oxidation [78]. Bacterial isolates, which were identified in vitro as sulfur-oxidizers, enhance sulfur availability and uptake in canola [79]. Genes involved in both sulfur metabolism and transport were identified in *P. dispersa* C2H3, *R. terrigena* C6G7, and *Ochrobactrum* sp. C6C9. *Ochrobactrum* sp. have previously been reported to metabolize sulfur [80,81]. Although sulfur is not often a component of commercial greenhouse fertilizers, sulfur deficiencies cause leaves to be a yellowish or light green color. Increased sulfur uptake in plants treated with *R. terrigena* C6G7 and *Ochrobactrum* sp. C6C9 may have contributed to their higher color ratings.

PGPR can produce siderophores that chelate iron and make it available for uptake by plants [82]. All of the top six strains except *Ochrobactrum* sp. C6C9 contained at least one biosynthetic gene cluster (BGC) encoding for siderophores. Patel et al., [61] found that a strain of *P. dispersa* isolated from the rhizosphere of mung bean plants produces siderophores and increases iron content in plants, and Singh et al. [76] reported that *P. oryzihabitans* isolated from maize produces siderophores in vitro.

Many PGPR can influence plant nutrition by modifying the root system architecture. These changes result in increased numbers and length of lateral roots and root hairs, allowing for the increased uptake of mineral nutrients and water [83]. Changes to plant root systems can be the result of PGPR modulating the balance of phytohormones (primarily auxin and cytokinins) in the host plant [39]. The most well characterized auxin produced by PGPR is IAA, and it is usually synthesized from Trp via the indolepyruvic acid pathway [83]. The genomes of *P. dispersa* C2H3 and *R. terrigena* C6G7 contained indole-3-pyruvate decarboxylase, the key enzyme in this pathway, but only strain C2H3 also contained the Trp biosynthesis operon. A strain of *P. dispersa* isolated from chickpeas (*Cicer arietinum*) has been reported to produce IAA [84,85]. Although the production of cytokinins has been reported in multiple genera of PGPR, including *Pseudomonas*, the genes involved in this synthesis have not been experimentally validated [83].

Many PGPR produce ACC deaminase, which degrades ACC and reduces ethylene production in plants [40,41]. Decreasing the production of ethylene in plant roots can reduce the inhibitory effects of ethylene on root growth [86,87,88]. Genome sequence data confirmed that *C. zhejiangensis* C7B12 and *P. oryzihabitans* C4H3 contained the ACC deaminase gene (*acdS*) (Table 3). Both strains were originally selected from the greenhouse rhizospheric bacterial collection for ACC deaminase activity in vitro [46]. Similarly, an ACC deaminase-encoding strain of *P. oryzihabitans* isolated from the rhizosphere of stressed pineapple plants (*Ananas comosus*) was identified based on its ability to use ACC as its sole nitrogen source [89].

Ornamental plant performance is based on visual attributes that include the flowers, the color and architecture of the vegetation, the lack of damage, and size [4,5]. Since PGPR selection has focused on field crops, plant performance measures that reflect a high-quality field crop, such as yield, are used to screen potential PGPR. For field trials of pearl millet treated with PGPR, growth parameters included plant height, yield, and number, as well as the length and girth of the cob [48], and for greenhouse and field trials of cotton treated with PGPR, plant biomass and yield parameters were measured [45]. In our greenhouse validation trial, many parameters to assess flowering, vegetative growth, and vegetative quality (Table 2, Figure 3, Figure 4, Figure 5, Figure 6, Figure 7, Figure 8 and Figure 9) were measured over time to identify the best parameters for capturing changes in plant performance for greenhouse ornamental crops after bacterial applications and to develop recommendations for future experiments.

Looking within the flowering performance category, some strains increased the flower number (i.e., *Microbacterium* sp. C5A9 and *P. putida* C4E12) but were not identified as a top performer for flower DW (Table 2). Flower size is an important factor for the overall quality of ornamental plants, and we do not want PGPR applications to decrease the size of individual flowers. Since it is often impractical to weigh all the individual flowers in these experiments, it is important to include both flower number and total flower DW to assess the impact on flowering. The final growth index measurement (week 3), canopy cover, plant architecture rating, and shoot DW all provided information on the vegetative growth at the end of the experiment, but the same bacterial strains were not identified for all these parameters. For example, *Pseudomonas oryzihabitans* C4H3 was identified for an increase in the growth index in week 3 and canopy cover, but not for the plant architecture rating or shoot DW. For greenhouse-grown ornamentals, the shape and denseness of the plant is an important plant performance characteristic. A larger-sized plant, indicated by a larger growth index or shoot DW, is not always the goal. Canopy color and the plant architecture rating provide information on the denseness or sparseness of the plant’s vegetation. These results demonstrate that using diverse vegetative growth parameters can provide different information about plant performance after the application of PGPR.

It was expected that the color quality rating and the soil-plant analysis development (SPAD) index measurements would identify the same bacterial strains. This was not true for *P. oryzihabitans* C4H3, which was identified for the highest color rating but was not identified as a top performer in any week for SPAD index (Table 2). *Herbaspirillum* sp. C7C2 was identified as a top performer for its improved SPAD index in week 2 and week 4 but not for any other parameters, including the color quality rating. The color quality rating considered the visual color quality of the shoot vegetation, while the SPAD index expressed the relative chlorophyll concentration of the single leaf measured. SPAD readings have been correlated with leaf nitrogen concentration, but values are highly dependent on the leaf selected, especially under nutrient-limiting conditions [90,91]. Since vegetation color quality is important in ornamental crop production [5], the SPAD index and color quality rating are both good parameters to consider when studying crop performance.

Measuring multiple parameters within the different performance categories allowed for the identification of overall top-performing strains, but also allowed for the identification of strains that improved plant performance in only certain categories (i.e., flowering, vegetative growth, or vegetative quality) (Table 2). This may suggest that different bacteria improve plant performance via different modes of action. While most of the performance parameters in this study were measured at the end of the experiment 4 weeks after transplant, the weekly growth index and SPAD measurements allowed us to determine when changes in plant performance were first observed as a result of specific bacterial treatments. *Pseudomonas corrugata* C8D4 was identified as a top performer based on its higher SPAD index at week 4 and higher growth index at week 3 (Table 2). Later improvements in plant performance may be due to the need for root colonization, and the subsequent timing required for the plant to benefit from increased nutrient availability and uptake resulting from this relationship. Bacterial colonization of the plant roots depends on the root exudates from the plant, as well as the bacteria’s preference for certain metabolites produced by the plant [27,92]. In contrast, *R. terrigena* C6G7 had higher SPAD values in weeks 2 and 3, but not in week 4. Early, but inconsistent, improvement in wheat plant performance after PGPR application with no colonization was observed by de Freitas [93], who attributed this to possible plant growth hormone production rather than increased nutrient uptake. The bacterial production of secondary metabolites can also result in improved plant yield and growth [94]. It has been previously reported that a strain of *R. terrigena* was found to produce hormones (e.g., IAA), amino acids (e.g., lysine), organic acids (e.g., malonic acid), and antioxidant enzyme activities (e.g., superoxide dismutase) that can increase plant performance [95].

Some strains were identified as top performers in several parameters within a single performance category, and this type of information may become useful when trying to formulate bacterial consortia to optimize plant growth promotion. *Herbaspirillum* sp. C9C3 was a top performer in the category of flowering in both the flower number and flower DW parameters (Table 2). Many species within the *Herbaspirillum* genus have been found to colonize plants and improve plant performance [96]. One *Herbaspirillum* sp. strain was identified as a nitrogen fixer for wild rice (*Oryza officinalis*) [97], and strains from the *Herbaspirillum* genus were selected for the ability to solubilize phosphorus, increasing rice (*Oryza sativa*) grain yield [98]. Knowing that the plant parameter is improved by a specific strain and the timing of these growth improvements can lead to the formulation of bacterial consortia that contain individual bacteria with different modes of action [23]. A PGPR consortium containing a nitrogen fixer, *Azospirillum brasiliensis* or *Azotobacter chroococcum*, along with a phosphorus solubilizer, *Bacillus subtilis*, and a potassium solubilizer, *Frateuria aurantia,* increases plant growth, yield, quality, and leaf nutrient content when applied to tobacco (*Nicotiana tabacum*) plants receiving only 75% the recommended dose of fertilizer [99].

In our study, although *C. zhejiangensis* C7B12 resulted in increased plant performance under low-fertility conditions, it was not the best strain when considering the color and chlorophyll content of the vegetation. When building a consortium of bacteria to improve plant performance, we should consider combining *C. zhejiangensis* C7B12 with *R. terrigena* C6G7, a strain that improved color quality and chlorophyll content (SPAD in weeks 2 and 3). The genome of *R. terrigena* C6G7 also encodes for genes involved in sulfur metabolism and transport that were not present in the *C. zhejiangensis* C7B12 genome and which may lead to improved sulfur uptake and greener leaves. Another potential combination would be *C. zhejiangensis* C7B12 and *P. oryzihabitans* C4H3, which was also selected for improved color quality. Future studies will consider these potential consortia based on what we have learned from the plant performance parameters and potential mechanisms of plant growth.

## 4. Materials and Methods

### 4.1. Bacteria Collections

The bacterial strains evaluated in this study were originally isolated from the rhizosphere of ornamental plants collected from 15 different Ohio and West Virginia greenhouse production facilities, as described by Nordstedt and Jones [46]. Ninety-four strains were originally selected from the greenhouse rhizosphere collection of 1056 bacterial isolates for the potential to alleviate water deficit stress [46]. These same 94 strains are now also being evaluated for the ability to improve plant performance under low-fertility conditions.

### 4.2. High-Throughput Greenhouse Trials

#### 4.2.1. Plant Material and Greenhouse Conditions

High-throughput greenhouse trials were conducted to determine if any of the 94 bacterial isolates could be used to improve plant performance when plants were grown with lower than optimal fertilizer applications (i.e., low fertility). The methods used for these greenhouse trials were similar to those of Nordstedt and Jones [46]. *Petunia* × *hybrida* ‘Picobella Blue’ (Syngenta Flowers, Gilroy, CA, USA) seeds were sown in plug trays with soilless, peat-based media (Pro-Mix PGX; Premier Tech Horticulture, Quakertown, PA, USA). Seedlings were fertilized at each irrigation with 50 mg L^−1^ N from 15N–2.2P–12.5K–2.9Ca–1.2Mg commercial water-soluble fertilizer (Jack’s Professional LX 15-5-15 Ca-Mg; JR Peters Inc., Allentown, PA, USA) and transplanted after 3 weeks into 6.4-cm pots of Pro-Mix PGX media. After transplanting, all plants were fertilized with Jack’s Professional LX at 25 mg L^−1^ N (3.6 mg L^−1^ P, 20.8 mg L^−1^ K, 6.7 mg L^−1^ Ca, and 3.3 mg L^−1^ Mg) from 15N–2.2P–12.5K–2.9Ca–1.2Mg commercial water-soluble fertilizer at each irrigation with reverse osmosis (RO) water. Total N (15.0%) was made up of 3.0% NH_4_ and 12.0% NO_3_. This fertilizer also provided ≤ 0.125 mg L^−1^ B, Cu, Fe, Mn, Mo, and Zn. The low-fertility treatment of 25 mg L^−1^ N was chosen based on previous experiments [42]. Petunias are typically fertilized with 100–150 mg L^−1^ N from water-soluble fertilizer at each irrigation during production [100,101]. Greenhouse temperatures were maintained at 25 °C ± 3 °C during the day and 19 °C ± 3 °C at night, with a 16 h photoperiod provided by supplementary lighting from high-pressure sodium and metal halide lamps (GLX/ GLS e-systems GROW lights, PARSource, Petaluma, CA, USA). Lighting was maintained between 250 μmol m^−2^ s^−1^ and 350 μmol m^−2^ s^−1^ at plant level.

#### 4.2.2. Experimental Design and Bacterial Application

The high-throughput greenhouse trials were set up as a randomized complete block design with two treatment replicates per block and a total of six blocks (*n* = 12). Due to the large number of isolates, the bacterial treatments were evaluated in three independent trials. The smaller pot size contributed to the high-throughput nature of the greenhouse trials by allowing for more efficient greenhouse space usage and less bacterial inoculum needed to saturate the peat-based media, while still maintaining 12 replicates per treatment. The first bacterial treatment was applied the day after transplanting and then weekly until harvest. To prepare the bacterial inoculum, single colonies were used to inoculate liquid Luria–Bertani (LB) medium, and then grown for 16 h at 28 °C in a shaking incubator at 250 rpm. The optical density (OD) was then measured, and LB medium was used to adjust the cultures to an OD_595_ = 0.8. The cultures were then diluted 1:100 with RO water, and 40 mL of each treatment was applied as a drench to the peat-based media. In each trial, the plant-growth-promoting bacteria *Pseudomonas putida* UW4+ and its ACC deaminase-deficient mutant (UW4−) [40,88,102] were used as checks, because UW4+ has been reported to promote plant growth under abiotic stress [103,104,105]. A negative control, sterile LB medium diluted 1:100 with RO water, was included in each trial.

#### 4.2.3. Data Collection

Plants were evaluated 6 weeks after transplant to measure plant performance. The combined number of open flowers plus buds showing color, referred to as flower/bud number hereafter, were counted on each plant. The shoots (leaves and stems without the flowers) were harvested, placed in paper bags, dried in a forced air oven (49 °C), and weighed to determine shoot DW.

### 4.3. Taxonomic Classification of Top-Performing Bacterial Isolates

Genomic DNA was extracted from each bacterial isolate selected in the high-throughput greenhouse trials using the Quick-DNA Bacterial Miniprep Kit (Zymo Research, Irvin, CA, USA) following the manufacturer’s instructions. Whole-genome sequencing of isolates C7B12, C2H3, and C9C3 was conducted at CoreBiome, Inc. (St. Paul, MN, USA) as originally described by Nordstedt and Jones [46]. DNA sequencing libraries for the remaining isolates were prepared using the Nextera DNA Flex Library Preparation Kit (Illumina, San Diego, CA, USA) according to the manufacturer’s instructions. Libraries were sequenced using the Illumina iSeq 100 (Illumina, San Diego, CA, USA) using paired-end 1 × 150 reads. Sequence quality was checked using FastQC (Babraham Institute, Cambridge, UK) and sequences were assembled into contigs using SPAdes (v 3.14.0) (Center for Algorithmic Biotechnology, St. Petersburg, Russia). Prokka (v 1.14.6) (Victorian Bioinformatics Consortium, Melbourne, Australia) was used to annotate genes using the contig assemblies. The Microbial Genome Atlas (MiGA) was used for taxonomic classification by computing the average nucleotide identity (ANI) of the query sequence to the NCBI prokaryotic genome database [106]. Bacterial strains were determined to be the same species as those in the database when sharing an ANI greater than or equal to 94%. In cases where less than 94% ANI was shared, isolates were determined not to have a species-level match within the database and were designated with ‘species’ (i.e., *Ochrobactrum* sp.) [107] (Table 1).

### 4.4. Greenhouse Validation Trial with Low-Fertility Conditions

#### 4.4.1. Plant Material and Greenhouse Conditions

The 14 top-performing strains selected from the high-throughput greenhouse trials and identified by whole-genome sequencing were evaluated in the greenhouse validation trial using *Petunia* × *hybrida* ‘Picobella Blue’ (Syngenta Flowers). Seeds were sown in plug trays in Pro-Mix PGX (Premier Tech Horticulture, Quakertown, PA, USA), grown for 4 weeks in the greenhouse, and fertilized with 50 mg L^−1^ N from 15N–2.2P–12.5K–2.9Ca–1.2Mg water-soluble fertilizer (Jack’s Professional LX; JR Peters Inc., Allentown, PA, USA) at each irrigation. The seedlings were then transplanted into 11.4-cm plastic pots containing Pro-Mix PGX to simulate common production practices. The greenhouse was maintained at the same environmental conditions described for the high-throughput greenhouse trials. Beginning at transplant, all plants receiving bacterial treatments and the negative control plants were irrigated with Jack’s Professional LX 25 mg L^−1^ N from 15N–2.2P–12.5K–2.9Ca–1.2Mg commercial water-soluble fertilizer as described for the high-throughput greenhouse trials to induce low-fertility conditions. Plant growth parameters from bacterially-treated plants were compared to the negative control plants (no bacterial treatment), which were also fertilized with 25 mg L^−1^ N. To compare the performance of the bacterially-treated plants fertilized with only 25 mg L^−1^ N to plants receiving higher rates of fertilizer, four positive controls (no bacterial treatment) fertilized with either 50, 75, 100, or 150 mg L^−1^ N were also included. The 150 mg L^−1^ N rate was considered the optimal fertilizer rate.

#### 4.4.2. Experimental Design and Bacterial Application

The experiment was organized as a randomized complete block design with 12 blocks and a single plant replicate per block (*n* = 12). In each block, the 14 bacterial treatments, a negative control, and four different positive controls were included for a total of 19 treatments per block. The first bacterial treatment was applied the day after transplanting and repeated weekly until harvest. The bacterial treatments were prepared by inoculating liquid LB medium with a single colony and growing the cultures for 16 h at 28 °C in a shaking incubator at 250 rpm. LB medium was used to adjust the cultures to an OD_595_ = 0.8. Cultures were then diluted 1:100 with RO water, and 120 mL of each treatment was applied as a drench to the soilless, peat-based growing media. This drench volume saturated the soilless growing media without leaching. The negative and positive control plants were drenched with the same volume of sterile LB medium diluted 1:100 in RO water.

#### 4.4.3. Measuring Plant Performance

Overall plant performance under low-fertility conditions was determined by measuring multiple parameters within three performance categories, which included flowering, vegetative growth, and vegetative quality. Plant performance was measured throughout production and as part of a destructive harvest at the end of the experiment at 4 weeks post-transplant. Parameters measured in the performance category of flowering included the time to first flower, the number of open flowers per plant, total flower DW, the number of buds per plant, and the total bud DW at harvest. Parameters measured in the performance category of vegetative growth included weekly growth index, final canopy cover, plant architecture rating at harvest, and shoot DW at harvest. Parameters measured in the performance category of vegetative quality included weekly SPAD measurements and a color quality rating at harvest.

**Flowering parameters.** Time to first flower was determined for each plant as the number of days from transplanting to the first open flower. The total number of buds and open flowers were counted separately for each plant 4 weeks after transplant. The buds and flowers were then placed in separate paper bags, dried in a forced air oven (49 °C), and weighed to determine the bud and flower DWs.

**Vegetative growth parameters.** The growth index was used to assess vegetative growth weekly from transplanting (week 0) to week 3. The two perpendicular widths and the height of the plant from the media surface to the top of the plant were used to calculate the growth index [108].

$$growth\;index=\;\frac{height+\left(width\;1+width\;2\right)/2}{2}$$

After the buds and flowers were removed from the plant, canopy cover was measured using an automatic color threshold classification image analysis tool (Canopeo, Mathworks, Inc., Natick, MA, USA) [109]. The distance between the plant and the camera was kept consistent when taking these photos. The plant architecture was rated at harvest using the following scale: 1—vegetation did not cover the pot with ~40% of soilless growing media visible; plant appeared stunted; 2—vegetation did not cover the pot with ~25% of soilless growing media visible; 3—vegetation covered pot but some media was visible; 4—vegetation covered pot and vegetation was leggy; 5—vegetation covered pot fully; vegetation was not leggy. Shoots were harvested, placed in paper bags, dried, and weighed to determine the final shoot DW.

**Vegetative quality parameters.** Relative chlorophyll concentration was measured weekly from transplanting (week 0) to week 4 with a soil-plant analysis development (SPAD) meter (SPAD-502, Konica Minolta Sensing, Inc., Osaka, Japan). Measurements were taken on a single leaf per plant using the first fully expanded leaf. The color quality rating was measured using the following scale: 1—interveinal chlorosis on five or more leaves; 2—yellow-colored leaves with four or fewer leaves with interveinal chlorosis; 3—yellow-colored leaves with no interveinal chlorosis; 4—good green vegetation with no discoloration.

### 4.5. Statistical Analysis

#### 4.5.1. High-Throughput Greenhouse Trials

Statistical analyses were performed in R version 4.0.2 (R Foundation for Statistical Computing, Vienna, Austria). Data collected from the three trials were analyzed separately. Flower/bud number and shoot DW were used to select the top-performing strains from each trial. Flower/bud number was analyzed using a Poisson regression model and quasi-Poisson when appropriate [110], and shoot DW was analyzed with lme4 [111] using a linear mixed-effects model. The estimates of treatment effect were ranked compared to the negative control for each parameter. The top 10% of the total strains were then selected from this ranking for each parameter.

#### 4.5.2. Greenhouse Validation Trial

All parameters were compared to the negative control to determine the bacterial treatments that improved plant performance. The days to first flower, bud number, and flower number were all analyzed as count data using a Poisson regression model [110]. Growth index, SPAD index (leaf chlorophyll concentration), canopy cover, and all DWs were analyzed using a fitted linear mixed-effects model with lme4 [111], and a square root transformation was used when appropriate. Bacterial treatments were considered fixed effects and block was a random effect. The week 0 growth index and SPAD index, taken at the time of transplanting, were used as covariates for the remaining weekly growth index and SPAD index measurements. The *F*-test was considered significant at α = 0.100, and all treatments were compared to the negative control with Dunnett’s comparison (*p* ≤ 0.100) using the R package multcomp [112]. The plant architecture and color quality ratings were analyzed using the Kruskal–Wallis rank sum test [110] followed by Dunn’s test of multiple comparisons with the R package FSA [113]. Strains were selected based on the individual ratings after Dunn’s test that had higher quality ratings compared to the negative control (*p* ≤ 0.100).

All parameters, except for the days to first flower, were used to select the overall top-performing strains with the potential to increase plant performance under low-fertility conditions. Fixed-effect estimates of treatment effects for bud and flower number, growth index, SPAD index (leaf chlorophyll concentration), canopy cover, and all DWs were used to rank the treatments compared to the negative control for each parameter. The z-score was used for the plant architecture and color quality ratings to rank the treatments. The top ~25% of bacterial strains were selected from each parameter. To select the overall top-performing strain, each time the bacterial treatment appeared in the top strains for a parameter, it received one point. These points were then added together to calculate the overall score to select the overall top-performing strains.

### 4.6. Identification of Genes Involved in Putative Plant-Growth-Promoting Mechanisms

The annotation files for the top six bacterial strains were searched manually to predict bacterial genes involved in plant growth promotion. Targeted mechanisms included nitrogen and sulfur metabolism and transport; phosphate and zinc solubilization and transport; siderophore production; and phytohormone synthesis and degradation (see Table 3). AntiSMASH (v 5.0) was used to identify biosynthetic gene clusters (BGCs) [114] encoding for the production of siderophores (enterobactin, ornibactin, turnerbactin, desferrioxamine, and pyoverdin) putatively involved in increasing iron availability.

## 5. Conclusions

The high-throughput and greenhouse validation trials were successful in identifying bacterial strains that could improve plant performance in greenhouse production systems under low-fertility conditions. Plants treated with *Caballeronia zhejiangensis* C7B12, a species that has not previously been described as a PGPR, not only showed improved performance compared to negative control plants grown with the same fertilizer rate (25 mg L^−1^ N), but their plant growth and quality were similar to that of plants treated with 50 or 75 mg L^−1^ N. The second- and third-tier overall top-performing strains *P. dispersa* C2H3, *P. oryzihabitans* C4H3, *Curtobacterium* sp. C2H10, *R. terrigena* C6G7, and *Ochrobactrum* sp. C6C9 were identified as overall top strains based on smaller or fewer improvements in plant performance (i.e., flowering, vegetative growth, and vegetative quality), but these also show potential for use as PGPR. Most of the species identified as overall top strains have shown promise as PGPR in other crops, but not yet in greenhouse ornamentals under low-fertility conditions. The specific methodologies for the high-throughput greenhouse trials, greenhouse validation trial, data analysis, and strain selection provide a pipeline for the identification of new PGPR based on the improvement of desired plant performance parameters [23]. Various plant performance parameters were identified in the greenhouse validation trial that can be used for the future evaluation of bacterial isolates in ornamental greenhouse crop production. The different parameters measured in the greenhouse validation trial, along with the genomic analysis, provide insights into the putative mechanisms for growth promotion and will aide in the identification of bacteria than can be used as part of consortia that will optimize the desired plant responses. The use of PGPR-containing biostimulants in greenhouse ornamental plant production will help growers achieve sustainability goals by allowing for the production of high-quality plants with reduced chemical inputs. There is a growing global interest in the use of PGPR-containing biostimulants for many different crops in field and greenhouse production [16], and this work adds to that body of knowledge targeting the production of greenhouse-grown ornamental crops in peat-based growing media.

## Figures and Tables

**Table 1:** Top 15 bacterial strains selected from the high-throughput greenhouse trials.

					Parameter Selected for	
Bacterial Strain	Taxonomic Classification	Accession No. ^1^	Plant Origin ^2^	GH Facility Origin ^3^	Flower/Bud Number ^4^	Shoot DW ^4^
C7B12	*Caballeronia zhejiangensis*	SAMN14930932	*Plectranthus scutellarioides* ‘Electric Lime’	1	X	X
C4E8	*Pseudarthrobacter equi*	SAMN14930920	*Petunia* × *hybrida* ‘Peppy Blue’	2	X	
C6G7	*Raoultella terrigena*	SAMN14930926	*Plectranthus scutellarioides* ‘Vino’	3	X	
C4E12	*Pseudomonas putida*	SAMN14930924	*Plectranthus scutellarioides* ‘Vino’	4	X	
C2H10	*Curtobacterium* sp.	SAMN14930927	*Plectranthus scutellarioides* ‘Kingswood Torch’	5	X	
C4B10	*Pseudomonas putida*	SAMN14930922	*Catharanthus roseus* ‘Titan Lilac’	2	X	
C7C8	*Herbaspirillum* sp.	SAMN14930928	*Plectranthus scutellarioides* ‘Dark Star’	1		X
C8D4	*Pseudomonas* *corrugata*	SAMN14930921	*Plectranthus scutellarioides* ‘Fishnet Stockings’	6		X
C9C3	*Herbaspirillum* sp.	SAMN14854762	*Plectranthus scutellarioides* ‘Colorblaze Marooned’	7		X
C7C2	*Herbaspirillum* sp.	SAMN14930931	*Zinnia elegans* ‘Magellan’	8		X
C2H3	*Pantoea dispersa*	SAMN14930933	*Zinnia elegans* ‘Dreamland Coral’	9	X	
C3B1	Unknown	NA	*Plectranthus scutellarioides* ‘Wild Lime’	5	X	
C6C9	*Ochrobactrum* sp.	SAMN14930923	*Plectranthus scutellarioides* ‘Vino’	3		X
C5A9	*Microbacterium* sp.	SAMN14930925	*Pelargonium* × *hortorum* ‘Calliope Dark Red’	9		X
C4H3	*Pseudomonas oryzihabitans*	SAMN14930929	*Plectranthus scutellarioides* ‘Colorblaze Apple Brandy’	2		X

^1^ The accession no. is given for each strain (except C3B1) and can be found on the NCBI database with Bioproject no. PRJNA631210 or PRJNA633025. ^2^ Plant origin is the ornamental plant in which the bacterial strain was originally isolated. ^3^ The greenhouse (GH) facility origin is the production facility where the plant originated. Plants with the same number are from the same greenhouse facility. ^4^ Bacteria were selected for enhancing flower/bud number (number of flowers and buds on the plant at harvest) and/or shoot DW (dry weight).

**Table 2:** A summary of the top-performing bacterial strains in each performance parameter in the greenhouse validation trial with *Petunia* × *hybrida* ‘Picobella Blue’.

	Flowering ^1^				Vegetative Growth					Vegetative Quality				
Strain	Bud	Flower	Bud DW (g)	Flower DW (g)	GI ^2^ Week 2	GI Week 3	Canopy Cover ^3^	Plant Archt. ^4^	Shoot DW ^1^ (g)	SPAD ^5^ Week 2	SPAD Week 3	SPAD Week 4	Color Rating ^6^	Overall Score ^7^
C7B12	1	1	1	1	1	1	1	1	1		1	1		11
C2H3	1	1	1	1	1	1	1	1	1					9
C4H3	1		1			1	1						1	5
C2H10	1		1	1			1		1					5
C6G7	1								1	1	1		1	5
C6C9	1									1	1		1	4
C4B10										1	1	1		3
C5A9		1								1	1			3
C8D4						1						1	1	3
C9C3		1		1									1	3
C7C2										1		1		2
C4E12		1												1
C4E8													1	1
C7C8														0

^1^ The bud and flower number were counted and the bud, flower, and shoot dry weight (DW) were measured at harvest (4 weeks after transplant). ^2^ The weekly growth index (GI) was calculated from the height and two perpendicular widths. ^3^ Percent canopy cover was measured at plant harvest using an automatic color threshold classification image analysis tool. ^4^ Plant architecture ratings (plant archt.) on a 1 (vegetation did not cover the pot with ~40% of soilless substrate visible; plant appeared stunted) to 5 (vegetation covered pot fully; vegetation was not leggy) scale was measured at harvest. ^5^ A soil-plant analysis development (SPAD) meter was used to measure the relative chlorophyll concentration (SPAD index) weekly. ^6^ Color quality ratings on a 1 (interveinal chlorosis on five or more leaves) to 4 (good green vegetation with no discoloration) scale was measured at harvest. ^7^ A “1” is used to indicate in which parameters each bacterial strain was identified as the top-performing strain. The overall score is the summation of each column, giving a total number of parameters for which the strain was identified.

**Table 3:** Genes related to plant performance (i.e., growth promotion and stress tolerance) identified in the overall top-performing bacterial strains.

			Bacterial Strains ^1^					
Putative Plant Growth Promotion Property	Gene Function	Gene Name	C7B12	C2H3	C4H3	C2H10	C6G7	C6C9
Nitrogen metabolism and transport	Ammonium transport	*amtB-glnK*	x	x	x		x	
	Nitrate and nitrite transport	*nrtABC*						
	Nitrate and nitrite transport	*narK*	x				x	
	Nitrogen metabolism	*glnGL*	x		x			x
	Nitrite reduction	*nasDEF*						
	Nitrogenase enzyme	*nifDHK*						
Phosphate solubilization and transport	Pyrroloquinoline quinone synthase	*pqq*	x	x	x			x
	Glucose dehydrogenase	*gdh*	x	x	x			x
	High affinity phosphate transporter system	*pstABCS*	x	x	x		x	
Sulfur metabolism and transport	Sulfate ABC transporter	*cysPWAT-sbp*		x			x	x
	Sulfur metabolism	*cysND*	x	x	x		x	x
	Sulfur metabolism	*cysC*		x			x	
	Sulfur metabolism	*cysHIJ*		x			x	
Siderophore production (BGCs ^2^)	Enterobactin synthesis						x	
	Ornibactin synthesis		x					
	Turnerbactin synthesis			x	x			
	Desferrioxamine synthesis					x		
	Pyoverdin synthesis			x				
Zinc solubilization and transport	Glyoxylate/hydroxypyruvate reductase B	*ghrB*	x	x	x	x	x	
	High affinity ABC zinc transporter system	*znuABC*		x	x			x
	Zinc export	*zitB*	x	x		x	x	
	Zinc export	*zntABR*		x			x	x
IAA ^3^ synthesis	Trp ^4^ biosynthesis operon	*trpABCDE*	x	x	x	x		
	Trp-specific importer	*mtr*		x			x	
	Indole-3-pyruvate decarboxylase	*ipdC*		x			x	
	Conversion of indole-3-acetaldehyde to IAA	*aldAB*						
	Conversion of Trp to IAA	*iaaHM*						
ACC ^5^ degradation	ACC deaminase structural gene	*acdS*	x		x			

^1^ Overall top-performing strains identified from the greenhouse validation trial: *Caballeronia zhejiangensis* C7B12, *Pantoea dispersa* C2H3, *Pseudomonas oryzihabitans* C4H3, *Curtobacterium* sp. C2H10, *Raoultella terrigena* C6G7, and *Ochrobactrum* sp. C6C9. ^2^ BGCs: biosynthetic gene clusters. ^3^ IAA: indole-3-acetic acid. ^4^ Trp: tryptophan. ^5^ ACC: 1-aminocyclopropane-1-carboxylate.

## Data Availability

The whole-genome sequence data for the 14 top-performing strains can be found in the NCBI database with accession no SAMN14930920–SAMN14930929 and SAMN14930931–SAMN14930933 (BioProject no PRJNA633025) except for *Herbaspirillum* sp. C9C3, which can be found under accession no SAMN14854762 (BioProject no PRJNA631210).
